# Genome-wide single-nucleotide polymorphism linkage analyses of quantitative rheumatoid arthritis phenotypes in Caucasian NARAC families

**DOI:** 10.1186/1753-6561-1-s1-s105

**Published:** 2007-12-18

**Authors:** Kimberly E Taylor, Wei Chen, Christopher I Amos, Lindsey A Criswell

**Affiliations:** 1 University of California, San Francisco, Rosalind Russell Medical Research Center for Arthritis, 374 Parnassus Avenue, Box 0500, San Francisco, California 94143, USA; 2 Department of Epidemiology, University of Texas MD Anderson Cancer Center, 1155 Pressler Street, Unit 1340, Houston, Texas 77030, USA

## Abstract

We applied nonparametric quantitative trait linkage analysis to two rheumatoid arthritis quantitative phenotypes, IgM rheumatoid factor (RF) and anti-cyclic citrullinated peptide autoantibody titer measurements, using 5700 genome-wide Illumina single-nucleotide polymorphism genotypes on 658 Caucasian North American Rheumatoid Arthritis Consortium families. Peak LOD scores for both quantitative traits were located in the human leukocyte antigen region 6p21 (15.8 and 13.8 for RF and anti-cyclic citrullinated peptide, respectively) followed by 11p12 (3.2 and 3.6). In addition, there were LOD scores of 3.2 on 2q32 for RF and 3.6 on 4q24 for anti-cyclic citrullinated peptide. The resulting linkage signals for both phenotypes are very similar to previous results for rheumatoid arthritis as a qualitative variable, with rheumatoid factor measurements being most closely aligned. Interestingly, anti-cyclic citrullinated peptide exhibits a stronger linkage peak on 2p14 than rheumatoid factor and rheumatoid arthritis, and stronger linkage on 4q24. Finally, we used ordered subset analyses to determine if sub-ranges of these two traits increased rheumatoid arthritis linkage signals; however, our analyses did not reveal significant effects of the quantitative traits on rheumatoid arthritis linkage signals in this population.

## Background

Rheumatoid arthritis (RA) is a chronic autoimmune disease with heterogeneous phenotypes exhibited among affected individuals. While it is known to have a strong genetic component, studies attempting to identify chromosomal regions influencing RA have had mixed results, except for the human leukocyte antigen (HLA) region of chromosome 6 [1]. It is thought that the difficulty in identifying RA linkage regions may be due in part to its phenotypic heterogeneity, i.e., subtypes of this disease may have different genetic etiologies.

Recently, a genome-wide single-nucleotide polymorphism (SNP) analysis of North American Rheumatoid Arthritis Consortium (NARAC) families [2] implicated regions 2q33 and 11p12 in addition to the HLA region. Here we apply quantitative trait analysis to this same set of SNPs (provided as the Genetic Analysis Worskhop 15 (GAW15) Problem 2 data set), in order to explore genetic differences related to these phenotypes, and to take advantage of the potential increase in power of quantitative versus categorical analyses. In particular, we consider IgM rheumatoid factor (RF) and anti-cyclic citrullinated peptide (anti-CCP) autoantibody titer measurements, both of which are associated with RA but with incomplete and different specificities for the disease.

## Methods

Families were selected for analysis that had at least two affected members with Illumina SNP genotyping and anti-CCP and/or RF titers. We also analyzed the Caucasian subgroup of families to limit genetic heterogeneity. PEDSTATS [3] was used to check for Mendelian inheritance errors, tabulate family structure, and evaluate Hardy-Weinberg equilibrium of the SNPs. MERLIN [4] was used for all linkage analyses (MINX, Merlin in X, for X chromosome analyses). Chromosomal positions were converted to centimorgans using the approximation of 1 Mb = 1 cM, which has been demonstrated to not significantly affect linkage signals [5]. Prior to analyses, unlikely genotypes were filtered by MERLIN, as described in online MERLIN documentation [4,6].

Because linkage disequilibrium (LD) of tightly linked loci can lead to artificial inflation of linkage scores in the presence of some missing parental genotypes [7], regions of interest were analyzed using the MERLIN LD modelling feature. An *R*^2 ^value of 0.1 was used to define high-LD clusters.

Both quantitative traits were truncated – RF values below 11 (16%) were set to 11, and anti-CCP values above 210 (8%) were set to 210 – because measurement of data in these ranges is not reliable (GAW15 Problem 2 notes). QT linkage analyses were performed using the MERLIN "--deviates" option because both traits are not normally distributed and the NARAC sample is selected (in particular to contain multi-case RA families). This option necessitates specification of a population mean; in the absence of population data, we chose 11 as the mean for RF and 4.6 as the mean for anti-CCP, as done previously [8].

In order to directly compare the linkage results for RF and anti-CCP in regions of interest, we repeated the LD-modelled analyses using the subset of affected subjects having measurements for both quantitative traits.

Finally, anti-CCP and RF titers were evaluated for their influence on RA linkage on chromosomes 1 to 22 using the FLOSS (flexible ordered subset analysis) implementation [9] of ordered subset analysis [10]. The mean trait value of the family and the maximum difference among affected family members were both considered as ordering parameters to determine an optimal range of families for RA linkage evidence. FLOSS uses a Monte Carlo permutation test to compare the maximum ordered subset linkage score for this optimal set with the maximum obtained for random orderings of the families [9].

## Results

A total of 1419 affected individuals from 658 families met our selection criteria, with 1415 having RF titers and 1341 having anti-CCP titers. Most families (*n *= 580) had a single sib pair meeting this criterion. In addition, there were 60 families with 3 affected members, 12 families with 4, 5 families with 5, and 1 family with 6 affected members. PEDSTATS reported a total of 814 sib pairs, 34 half-sib pairs, 11 cousins, 8 parent-child pairs, and 27 avuncular pairs. Of the affected individuals, 40% had one parent genotyped, and 10% had both parents genotyped.

PEDSTATS did not encounter any Mendelian errors in the available genotypes of these families. There were no HWE *p*-values below 0.0001, which we had chosen as the cut-off at which to remove SNPs; there was only one SNP (on chromosome 3) with *p *< 0.001. Filtering of unlikely genotypes by MERLIN eliminated 5254 person-markers, 0.05% of the available genotypes.

With the resulting pedigrees, MERLIN (--deviates) was first run for all of chromosomes 1 through 22. MINX was used to analyze the X chromosomes. Then all chromosomes having peak LOD score > 3 were rerun with MERLIN's LD cluster modeling (*R*^2 ^value = 0.1). The results of these peak locations with and without LD modeling are shown in Table 1. As in Amos et al. [2], the most substantial decrease when modeling LD was on chromosome 21. LOD scores over 3 with LD cluster modelling are in bold.

**Table 1:** Peak LOD scores and locations with/without LD clusters.

		Without LD clusters		With LD clusters	
			
Trait	Chromosome	Peak LOD	Locations (kb)	Peak LOD	Locations (kb)
Anti-CCP	2	3.1	70859	2.8	66769, 67023
	4	4.4	104210	**3.6**	104353
	6	14.9	28965	**13.8**	32992
	11	4.4	39183, 39201	**3.6**	40486
	21	8.3	37442, 37455	2.4	40861
					
RF	2	3.2	194143	**3.1** (2.3^a^)	194143
	5	3.6	13709, 13735	2.6	13722
	6	18.4	28965	**15.8** (**14.4**^a^)	32992
	10	3.4	60473, 60475	2.6	75017
	11	4	39201	**3.2** (**4.2**^a^)	41024
	12	3.7	131968, 132017	1.6	22217
	19	4.5	63636, 63638	0.2	61193, 61823
	21	11.8	37433–37442	1.5	40861
	23-X	3.2	56386–56493	0.83	10450

^a^Using same set of subjects as anti-CCP analyses

**Figure 1:**
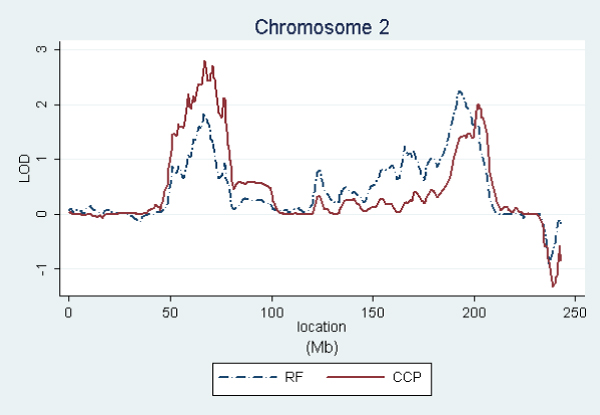
RF and anti-CCP linkage on chromosome 2 for affected subjects having measurements for both traits.

**Figure 2:**
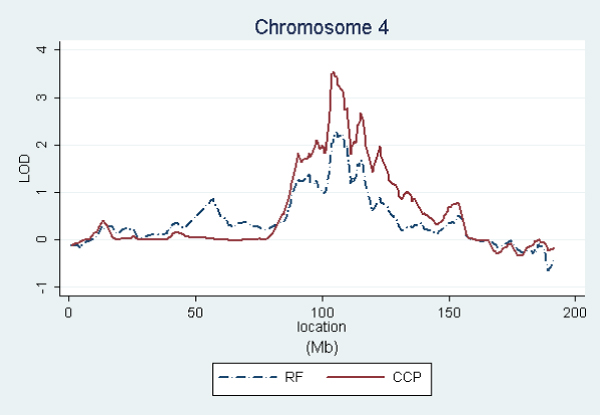
RF and anti-CCP linkage on chromosome 4 for affected subjects having measurements for both traits.

**Figure 3:**
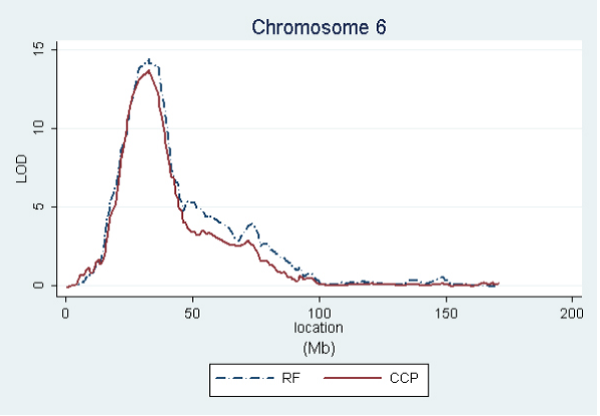
RF and anti-CCP linkage on chromosome 6 for affected subjects having measurements for both traits.

**Figure 4:**
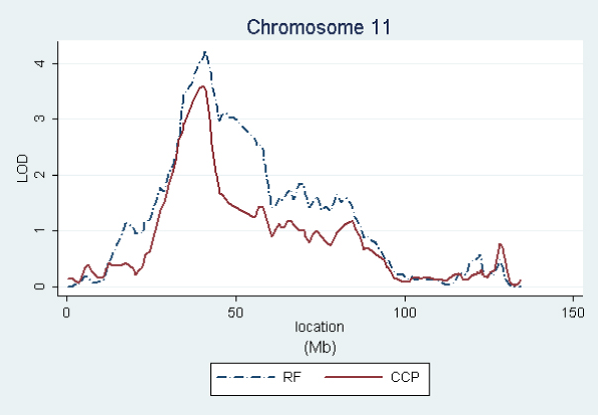
RF and anti-CCP linkage on chromosome 11 for affected subjects having measurements for both traits.

In order to graphically compare the linkage curves of RF and anti-CCP titers on chromosomes 2, 4, 6, and 11, we repeated these analyses using only the affected subjects having both measurements. This kept the same families for anti-CCP, but reduced RF from 658 to 609 families (see footnoted items in Table 1). Figures 1, 2, 3, 4 show the resulting linkage curves.

Finally, we explored whether there were RF or anti-CCP trait-ordered subsets [10] that produced better evidence for RA linkage using FLOSS [9]. We used two per-family trait measures, the mean trait value and the maximum trait difference. Using this data set, we did not observe significant effects of the quantitative trait subsets on RA linkage signals. The most significant result (*p *= 0.04) was for the effect of RF maximum difference on linkage to chromosome 17, but with consideration for multiple testing (two traits for 22 chromosomes) this result does not provide convincing evidence for a non-random association with RA linkage.

## Discussion

Our quantitative trait linkage results are similar to the RA linkage results using the same SNP set [2], with RF being most closely aligned to RA and anti-CCP exhibiting some interesting differences. For all three traits, the well established linkage peak in the HLA region on 6p21 extends far in the centromeric direction, extending into 6q and suggesting a possible secondary peak on 6q. A similar shape of lesser magnitude is exhibited by all three traits on chromosome 11p12. Linkage peak locations are also aligned on 4q24, but with the anti-CCP association being stronger (3.6 versus 2.3 using the same sample). On chromosome 2, RA and RF have the peak LOD score on 2q32–33, while the peak for anti-CCP is on 2p14. This difference in peaks is most pronounced in RA as described in Amos et al. [2]. Therefore, chromosome 2 may be interesting to explore further to understand the differences between RF and anti-CCP and their relationships to RA etiologies.

Differences in linkage evidence compared with an earlier microsatellite quantitative trait locus (QTL) analysis of a subset of these families [8] – in particular on regions 2p14, 2q32, 4q24, and 11p12 – are very similar to differences in these two scans seen in RA linkage [2]. For RA, differences were attributed to the SNP scan having 44% higher information content, a larger set of families, and the possible heterogeneity of non-Caucasians included in the microsatellite analyses [2]. Similarities between the RA and quantitative trait results are partially expected due to their correlation in the population, but are also addressed below.

The MERLIN deviates method was chosen for this analysis due to the non-normal trait distributions and because the trait sample was selected to only include RA subjects. We did not pursue variance-components [11] methods, including the Tobit VC method for censored data [12], because they assume normally distributed traits. However, when using the MERLIN deviates method, we have observed that single-point linkage scores for single sib pair families (88% of our selection) depend only on the direction of the quantitative trait from the mean. Because our selected RA sample is strictly above the RF population mean, and most are also above the CCP population mean, this reduces to RA linkage for a large portion of this data set. We believe that this partially explains the similarity in results between the RA and QTL linkage results for both our SNP analysis and the microsatellite analysis of [8]. We continue to investigate QTL methods that are appropriate for this selected non-normal data but can distinguish effects of the quantitative traits from RA.

Using ordered subset analyses, we did not identify any subset of families, based on quantitative trait ranges, which significantly increased the RA linkage signal. However, it is possible that this could be more successfully applied in a less selected population.

## Competing interests

The author(s) declare that they have no competing interests.
